# Comparison of Transjugular Intrahepatic Portosystemic Shunt in the Treatment of Cirrhosis With or Without Portal Vein Thrombosis: A Retrospective Study

**DOI:** 10.3389/fmed.2021.737984

**Published:** 2021-10-04

**Authors:** Hong-Liang Wang, Wei-Jie Lu, Yue-Lin Zhang, Chun-Hui Nie, Tan-Yang Zhou, Guan-Hui Zhou, Tong-Yin Zhu, Bao-Quan Wang, Sheng-Qun Chen, Zi-Niu Yu, Li Jing, Jun-Hui Sun

**Affiliations:** ^1^Hepatobiliary and Pancreatic Interventional Treatment Center, Division of Hepatobiliary and Pancreatic Surgery, The First Affiliated Hospital, Zhejiang University School of Medicine, Hangzhou, China; ^2^Zhejiang Provincial Research Center for Diagnosis and Treatment of Hepatobiliary Diseases, Hangzhou, China; ^3^Zhejiang Clinical Research Center of Hepatobiliary and Pancreatic Diseases, Hangzhou, China

**Keywords:** transjugular intrahepatic portosystemic shunt, cirrhosis, portal vein thrombosis, survival, shunt dysfunction

## Abstract

**Aim:** The purpose of our study was to conduct a retrospective analysis to compare the effectiveness of transjugular intrahepatic portosystemic shunts (TIPS) in the treatment of patients with cirrhosis with or without portal vein thrombosis (PVT).

**Methods:** We included a total of 203 cirrhosis patients successfully treated with TIPS between January 2015 and January 2018, including 72 cirrhosis patients with PVT (35.5%) and 131 without PVT (64.5%). Our subjects were followed for at least 1 year after treatment with TIPS. Data were collected to estimate the mortality, shunt dysfunction, and complication rates after TIPS creation.

**Results:** During the mean follow-up time of 19.5 ± 12.8 months, 21 (10.3%) patients died, 15 (7.4%) developed shunt dysfunction, and 44 (21.6%) experienced overt hepatic encephalopathy (OHE). No significant differences in mortality (*P* = 0.134), shunt dysfunction (*P* = 0.214), or OHE (*P* = 0.632) were noted between the groups. Age, model for end-stage liver disease (MELD) score, and refractory ascites requiring TIPS were risk factors for mortality. A history of diabetes, percutaneous transhepatic variceal embolization (PTVE), 8-mm diameter stent, and platelet (PLT) increased the risk of shunt dysfunction. The prevalence of variceal bleeding and recurrent ascites was comparable between the two groups (16.7 vs. 16.7% *P* = 0.998 and 2.7 vs. 3.8% *P* = 0.678, respectively).

**Conclusions:** Transjugular intrahepatic portosystemic shunts are feasible in the management of cirrhosis with PVT. No significant differences in survival or shunt dysfunction were noted between the PVT and no-PVT groups. The risk of recurrent variceal bleeding, recurrent ascites, and OHE in the PVT group was generally similar to that in the no-PVT group. TIPS represents a potentially feasible treatment option in cirrhosis patients with PVT.

## Introduction

Portal vein thrombosis (PVT) is an important complication of cirrhosis but is not common in the general population. However, in cirrhosis patients, the occurrence rate is ~10–25%, and this figure increases with the severity of cirrhosis (1–4). PVT can further aggravate portal vein hypertension and lead to repeated variceal bleeding or refractory ascites (5). PVT in cirrhosis may be associated with a reduction in the portal vein blood flow velocity, a high coagulation state, and vascular endothelial injury (6–8). Currently, anticoagulant therapy is recommended as the preferred treatment option for PVT, but anticoagulation is a challenging therapy in patients with liver cirrhosis given the well-recognized coagulation abnormalities (9). In addition, the occurrence of PVT is typically not evident, and most patients are complicated with portal hypertension (10, 11). At present, thrombosis exhibits different degrees of transformation into chronic thrombosis, and clinical treatment is very difficult. Transjugular intrahepatic portosystemic shunts (TIPS) can be used to establish a shunt between the hepatic vein and the portal vein to reduce portal vein pressure. TIPS improve portal vein blood flow and promote blood clot absorption and recanalization (12–15). Thus, the purpose of our study was to conduct a retrospective analysis to compare the effectiveness of TIPS in the treatment of patients with cirrhosis with or without PVT in our center.

## Materials and Methods

### Patients

This retrospective study was reviewed and approved by the ethics committee of The First Affiliated Hospital, Zhejiang University School of Medicine. Given its retrospective nature and the lack of a need to collect samples from patients, a waiver of written informed consent was applied. This study included all patients with cirrhosis (any etiology) with or without PVT characterized as non-neoplastic (no tumor vein invasion) according to criteria validated in previous studies (16) between January 2015 and January 2018 in our center. The exclusion criteria for our study included previous TIPS placement, missing clinical and demographic information, hepatocellular carcinoma, previous liver transplantation (LT), technical failure of TIPS, <12 months of follow-up, any active tumor at the time of PVT diagnosis, and incomplete baseline data. However, our study did not exclude patients with cavernomatous transformation of the portal vein. Patients were categorized according to whether they had PVT or not before TIPS. Patients were followed until the occurrence of end points, including death, LT, or the end of the study, in January 2018.

### TIPS Procedure

As previously described (17), TIPS procedures were performed by the same team of interventional radiologists who had >10 years of experience in TIPS procedures. With the exception of emergencies, all patients underwent routine computed tomography angiography (CTA) examination prior to the TIPS procedure to clearly visualize the anatomical relationship between the start (hepatic vein) and the end point (portal vein). After a successful puncture, bare stents (Boston Scientific, USA) plus a self-expandable polytetrafluoroethylene (ePTFE)-covered stent (GORE VIABAHN, USA), either 8 or 10 mm in diameter, were inserted primarily based on the data from the procedure that was performed. The portosystemic pressure gradient (PSG) was measured using the difference between the portal vein pressure and the right atrial pressure. If the PSG was not reduced below the target threshold (12 mmHg), balloon dilation was performed. TIPS revision using balloon dilatation or parallel TIPS was performed. Variceal embolization was based on post-TIPS portography using a metal coil (Cook, Bloomington, USA), glue (Guangzhou Baiyun, Guangdong, China), or a metal coil plus glue.

### Data Collection and Follow-Up

Clinical, epidemiologic, laboratory, and radiologic data were extracted from the medical records of the patients, including demographics, etiology of cirrhosis, previous splenectomy, history of diabetes, history of overt HE, and laboratory testing results (aspartate aminotransferase, alanine aminotransferase, albumin, international normalized ratio, platelet, hemoglobin, white blood cell, creatinine, and total bilirubin). Child–Pugh score, Child–Pugh class, model for end-stage liver disease (MELD) score, and Eastern Cooperative Oncology Group (ECOG) score were also calculated for each patient. The following TIPS outcomes were assessed in the entire cohort: duration of follow-up, indications for TIPS, refractory ascites, variceal bleeding (gastric plus esophageal), 90-day mortality, mortality (liver failure, multiorgan failure, gastrointestinal bleeding, hepatorenal syndrome, sepsis, cerebral hemorrhage, or unknown), stent (8 or 10 mm), overt hepatic encephalopathy (OHE), recurrent variceal bleeding, recurrent ascites, antiplatelet treatments (aspirin or aspirin plus dipyridamole), percutaneous transhepatic variceal embolization (PTVE) (coil, glue, or glue plus coil), portosystemic gradient before TIPS, portosystemic gradient after TIPS, LT, and shunt dysfunction. The presence of PVT was determined according to computed tomography (CT) or magnetic resonance imaging and confirmed by portal angiography at the time of TIPS creation. Follow-up visits were performed when patients presented to the follow-up clinic and were scheduled 1, 2, 3, and 6 months after TIPS and every 6 months thereafter. Clinical, laboratory, and liver CTA evaluations were performed at each visit, and the occurrence of any liver-related complications since the last visit was collected by the clinical research coordinator. Patients underwent follow-up until death, LT, or the end date of the study on January 31, 2018.

### Statistical Analysis

All continuous variables are presented as the mean ± SD, and categorical variables are expressed as counts and frequencies. Student's *t*-test or a chi-square test was used to compare the significant differences between groups where appropriate. Survival was calculated as the time from TIPS creation to the time of death, transplantation, or the last follow-up. Survival curves and the cumulative incidence of shunt dysfunction curves were estimated using the Kaplan–Meier method, and the differences were compared using the log-rank test. Risk factors associated with survival were explored using the Cox hazard multivariate regression model. To rule out the effect that splenectomy might have in our findings, we also performed the analysis excluding patients who had undergone splenectomy. We regard a two-tailed *p* < 0.05 as statistically significant. All the statistical analyses were conducted with R 3.5.0 (18).

## Results

### Baseline Characteristics

A total of 208 patients underwent TIP creation, but five patients experienced technical failure of TIPS, including two in the non-PVT group and three in the PVT group. Finally, a total of 203 patients who underwent TIP creation, including 72 patients with PVT, were included in our analysis. Table 1 presents the baseline characteristics of the patients and comparisons between the two groups. Age (*P* = 0.736) and sex (*P* = 0.424) were similar between the two groups. Hepatitis B, non-alcoholic steatohepatitis (NASH)/cryptogenesis, and liver disease caused by alcohol were identified as the most common causes of cirrhosis. The prevalence of hepatitis B infection, NASH/cryptogenesis, and liver disease due to alcohol consumption were generally comparable between the PVT and no-PVT groups [(58.3 vs. 52.7%, *P* = 0.594), (15.1 vs. 11.5%, *P* = 0.321), and (16.7 vs. 16.8%, *P* = 0.672), respectively].

**Table 1:** Baseline demographic and clinical characteristics of patients.

**Demographics**	**Overall (*N* = 203)**	**PVT (*N* = 72)**	**No-PVT (*N* = 131)**	***P*-value**
Male	141 (69.5.0%)	47 (65.3%)	94 (71.7%)	0.424
Age (mean ±SD) (years)	55.4 ± 10.8	55.2 ± 10.5	55.6 ± 11.4	0.736
**Etiology of cirrhosis**
Hepatitis B virus	111 (54.7%)	42 (58.3%)	69 (52.7%)	0.594
Hepatitis C virus	4 (1.9%)	1 (1.4%)	3 (2.1%)	0.421
Alcohol	34 (16.7%)	12 (16.7%)	22 (16.8%)	0.675
NASH/cryptogenic	26 (12.8%)	11 (15.3%)	15 (11.5%)	0.321
PBC/PSC	7 (3.4%)	1 (1.4%)	6 (4.6%)	0.257
Autoimmune	5 (2.5%)	3 (4.2%)	2 (1.5%)	0.132
Schistosome	10 (4.9%)	2 (2.8%)	8 (6.1%)	0.171
**Previous splenectomy**	27 (13.3%)	18 (25.0%)	9 (6.8%)	<0.001
**History of diabetes**	39 (19.3%)	11 (15.2%)	28 (21.4%)	0.262
**History of overt HE**	8 (3.9%)	2 (2.3%)	6 (4.6%)	0.324
**Laboratory parameters (mean** **±** **SD)**
AST (IU/l)	84 ± 212	86 ± 148	72 ± 256	0.232
ALT (IU/l)	65 ± 172	69 ± 124	62 ± 225	0.532
Albumin (g/dl)	34.3 ± 5.2	33.4 ± 4.4	34.6 ± 5.8	0.612
INR	1.3 ± 1.32	1.3 ± 1.22	1.3 ± 2.23	0.604
WBC	5.1 ± 5.1	5.8 ± 7.2	4.7 ± 3.2	0.231
PLT	76.1 ± 64.5	107.2 ± 82.4	65.4 ± 37.7	<0.001
HB	81.1 ± 24.7	76.4 ± 21.7	84.2 ± 256.2	0.021
Creatinine (mg/dl)	71.2 ± 31.2	67.4 ± 16.7	72.2 ± 35.5	0.325
Total bilirubin (mg/dl)	26.0 ± 19.45	24.6 ± 16.5	33.2 ± 22.4	0.432
**Child-Pugh score**	6.8 ± 1.4	6.8 ± 1.3	6.8 ± 1.4	0.632
**Child-Pugh class**				0.201
A	89 (43.6%)	29 (40.3%)	60 (45.8%)	
B	101 (49.5%)	40 (55.6%)	61 (46.6%)	
C	13 (6.4%)	3 (4.2%)	10 (7.6%)	
**MELD**	11.1 ± 3.20	10.9 ± 3.0	11.1 ± 3.3	0.324
**ECOG**	1.05 ± 0.27	1.04 ± 0.22	1.05 ± 0.23	0.703
**Stage of PVT (chronic)**	/	54 (75.0%)	/	
**Degree of PVT**	/		/	
Mural	/	12 (16.7%)	/	
Partial	/	54 (75.0%)	/	
Complete	/	4 (5.5%)	/	
**Extent of PVT**	/		/	
MPV alone	/	12 (16.7%)	/	
MPV + SMV	/	19 (26.4%)	/	
MPV + SV/splenectomy	/	32 (44.4%)	/	
MPV + SMV + SV/splenectomy	/	7 (9.7%)	/	

*NASH, non-alcoholic steatohepatitis; PBC, primary biliary cirrhosis; PSC, primary sclerosing cholangitis; HE, hepatic encephalopathy; ALT, alanine aminotransferase; AST, aspartate aminotransferase; INR, international normalized ratio; WBC, White Blood Cell; PLT, Platelet; HB, Hemoglobin; MELD, Model for End-stage Liver Disease; PVT, portal vein thrombosis. ECOG, Eastern Cooperative Oncology Group; MPV, main portal vein; SMV, superior mesenteric vein; SV, splenic vein*.

Comparing the two groups of patients, the PVT group had a greater proportion of patients with a history of splenectomy (25.0 vs. 6.8%, *P* < 0.001). History of diabetes and OHE did not significantly differ between PVT and no-PVT groups [(15.2 vs. 21.4%, *P* = 0.262) and (2.3 vs. 4.6%, *P* = 0.324)]. The following laboratory parameters were collected before TIPS: AST, ALT, albumin, INR, WBC, PLT, HB, creatinine, and total bilirubin. The PVT group had higher platelet counts (107.2 ± 82.4 vs. 65.4 ± 37.7, *P* < 0.001), but no obvious differences in other parameters were noted between the two groups. Child–Pugh, MELD, and ECOG scores were similar between the PVT and no-PVT groups [(6.8 ± 1.3 vs. 6.8 ± 1.4 *P* = 0.632), (10.9 ± 3.0 vs. 11.1 ± 3.3, *P* = 0.324), and (1.05 ± 0.27 vs. 1.05 ± 0.23, *P* = 0.703), respectively].

### Morbidity Following TIPS

**Figure 1:**
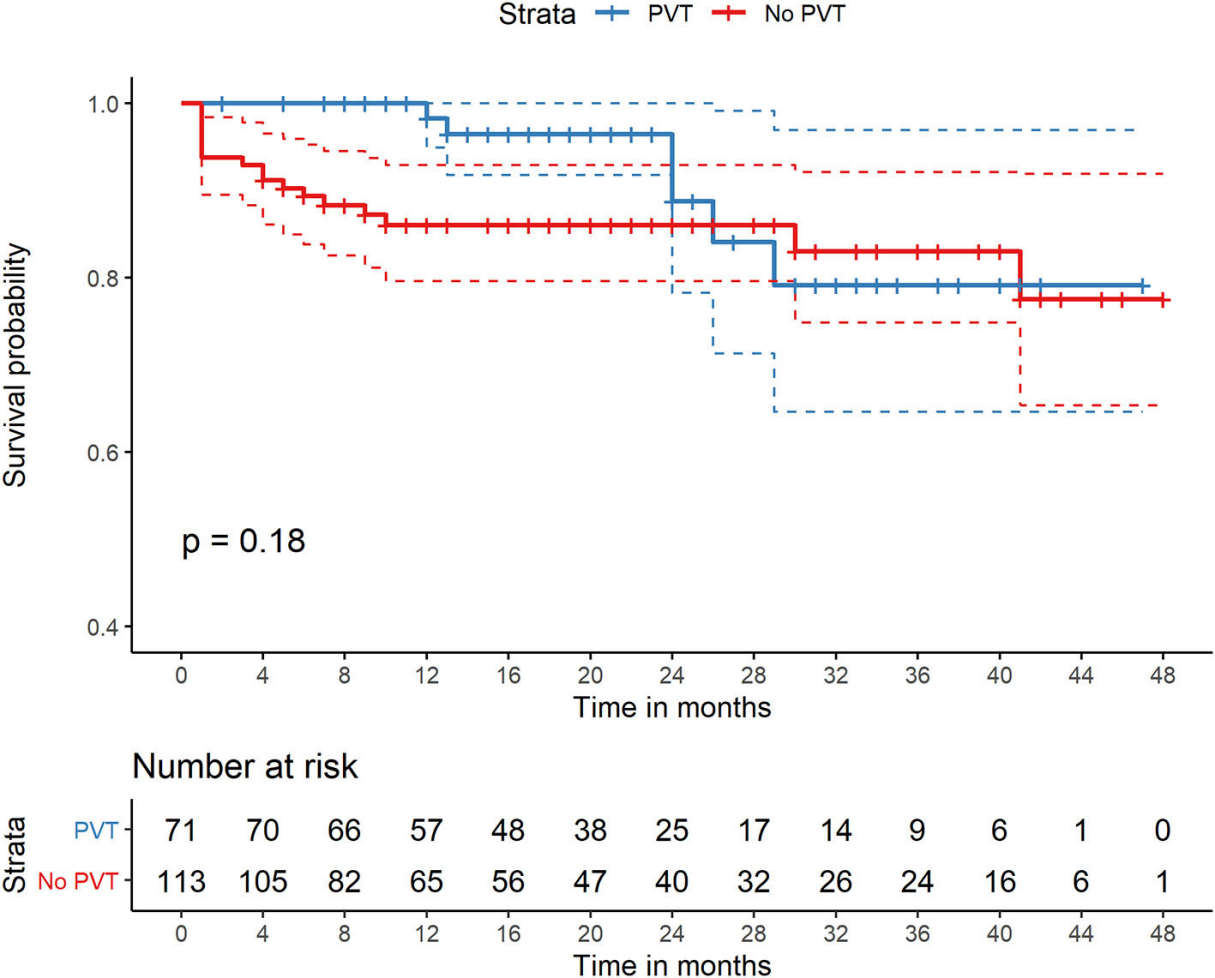
Comparison of survival of patients with PVT and without PVT who were all treated with TIPS. PVT, portal vein thrombosis; TIPS, transjugular intrahepatic portosystemic shunt.

During the mean follow-up time of 19.5 ± 12.8 months, 21 (10.3%) patients died. The cause of death included liver failure in eight patients (3.9%), multiorgan failure in six (2.9%), gastrointestinal bleeding in two (0.9%), hepatorenal syndrome in one (0.5%), sepsis in one (0.5%), cerebral hemorrhage in one (0.5%), and other conditions in two (0.9%). The cumulative incidence of death at 90 days and in the overall follow-up period was not significantly different between the PVT and no-PVT groups [(2.6 vs. 7.5%, *P* = 0.206) and (10.3 vs. 16.4%, *P* = 0.134), respectively] (Table 2, Figure 1). No significant difference was observed in the proportion of patients experiencing transplantation between the PVT and no-PVT groups (9.0 vs. 5.5%, *P* = 0.319).

**Table 2:** Outcomes of Transjugular intrahepatic portosystemic shunt in the Entire Cohort.

**Characteristics**	**Overall (*N* = 203)**	**PVT (*N* = 72)**	**No-PVT (*N* = 131)**	***P*-value**
**Duration of follow-up (month)**	19.5 ± 12.8	20.5 ± 10.2	18.8 ± 13.2	0.234
**Indications for TIPS**
Refractory ascites	23 (11.3%)	3 (4.2%)	20 (15.4%)	0.021
Variceal bleeding (Gastric + Esophageal)	184 (91.5%)	70 (97.2%)	114 (87.0%)	0.018
**90-day mortality**	8 (5.8%)	2 (2.6%)	6 (7.5%)	0.206
**Mortality**	21 (14.3%)	5 (10.3%)	16 (16.4%)	0.134
Liver failure	8 (3.9%)	2 (2.8%)	6 (4.5%)	
Multiorgan failure	6 (2.9%)	1 (1.4%)	5 (3.8%)	
Gastrointestinal bleeding	2 (0.9%)	1 (1.4%)	1 (0.7%)	
Hepatorenal syndrome	1 (0.5%)	0 (0.0%)	1 (0.7%)	
Sepsis	1 (0.5%)	0 (0.0%)	1 (0.7%)	
Cerebral hemorrhage	1 (0.5%)	0 (0.0%)	1 (0.7%)	
Unknown	2 (0.9%)	1 (1.4%)	1 (0.7%)	
**Diameter of stent**				0.622
8 mm	189 (92.6%)	68 (94.4%)	121 (92.3%)	
10 mm	14 (7.4%)	4 (5.6%)	10 (7.7%)	
**Overt hepatic encephalopathy**	44 (21.6%)	14 (19.4%)	30 (22.9%)	0.632
**Recurrent variceal bleeding**	34 (16.7%)	12 (16.7%)	22 (16.7%)	0.998
**Recurrent ascites**	7 (3.4%)	2 (2.7%)	5 (3.8%)	0.678
**Antiplatelet treatments**	114 (55.9%)	50 (69.4%)	64 (45%)	0.032
Aspirin	80 (39.2%)	36 (50.0%)	44 (30.1%)	0.021
Aspirin + Dipyridamole	34 (16.7%)	13 (18.1%)	21 (16.0%)	0.632
**PTVE**	152 (74.5%)	47 (65.3%)	105 (80.1%)	0.256
Coil	72 (35.3%)	25 (17.3%)	47 (36.3%)	0.782
Glue	22 (10.8%)	7 (9.7%)	15 (10.3%)	0.421
Glue + Coil	48 (23.5)	15 (20.8%)	33 (25.3%)	0.421
**Portosystemic gradient before TIPS (mmHg)**	25.2 ± 5.6	27.4 ± 6.2	24.1 ± 4.5	0.234
**Portosystemic gradient after TIPS**	10.5 ± 5.8	12.1 ± 6.2	9.5 ± 3.2	0.432
**Shunt dysfunction**	15 (7.4%)	7 (9.7%)	8 (6.1%)	0.214

*Indications for TIPS, 90-Day Mortality, Mortality, Diameter of stent, Overt hepatic encephalopathy, Recurrent variceal bleeding, Recurrent ascites, Antiplatelet treatments, PTVE, PSG before TIPS (mmHg), PSG after TIPS (mmHg), Liver transplantation, Shunt dysfunction compared between patients with and without PVT. OHE, Overt hepatic encephalopathy; PSG, Portosystemic gradient; PTVE, percutaneous transhepatic variceal embolization*.

Univariate analysis showed that age (per year increase), refractory ascites requiring TIPS, variceal bleeding (gastric plus esophageal) as an indication for TIPS, creatinine, MELD score, and ECOG score was associated with mortality risk. The Cox hazard multivariate regression model showed that only older age (hazard ratio (HR), 1.05; 95% CI, 1.04–1.09), higher MELD score (HR, 1.23; 95% CI, 1.08–1.41), and refractory ascites requiring TIPS (HR, 0.46; 95% CI, 0.32–0.65) were statistically significant predictors of mortality (Table 3). In addition, the usage of glue in PTVE (HR, 0.31 95% CI, 0.08–1.18) may improve overall survival compared with coil alone in PTVE (HR, 0.66 95% CI, 0.14–3.16).

**Table 3:** Factors associated with risk of mortality after Transjugular intrahepatic portosystemic shunt.

**Variable**	**Univariate analysis**				**Multivariate analysis**			
	**Hazard ratio^†^(95% CI)**			***P*-value**	**Hazard ratio^†^(95% CI)**			***P*-value**
Gender (male vs. female)	1.44	0.63	3.31	0.387	0.89	0.54	1.47	0.832
Age (per year increase)	1.05	1.01	1.09	0.010	1.04	1.00	1.09	0.032
**Antiplatelet treatments (yes vs. no)**	0.42	0.18	0.99	0.048	0.48	0.20	1.15	0.100
**History of ascites (yes vs. no)**	0.90	0.40	2.03	0.800				
**History of diabetes (yes vs. no)**	0.49	0.12	2.11	0.340				
**Previous splenectomy (yes vs. no)**	0.62	0.27	1.29	0.231				
Refractory ascites (yes vs. no)	0.48	0.28	0.81	<0.05	0.46	0.32	0.65	<0.05
**Varices bleeding (Gastric** **+** **Esophageal) (yes vs. no)**	0.71	0.31	1.63	0.414				
**PTVE (vs. no PTVE)**
Coil	0.83	0.32	2.17	0.708				
Glue	0.25	0.03	2.00	0.192				
Glue + Coil	0.86	0.31	2.43	0.784				
**Diameter of stent (8 vs. 10 mm)**	0.62	0.23	1.70	0.356				
Total bilirubin	1.00	0.97	1.02	0.817				
INR	1.71	0.32	9.06	0.530				
Creatinine	1.01	1.01	1.02	<0.001	1.01	1.00	1.02	0.067
PLT	1.00	0.99	1.01	0.647				
WBC	0.99	0.92	1.07	0.851				
HB	1.00	0.99	1.02	0.811				
Child	1.21	0.94	1.55	0.136				
MELD	1.15	1.05	1.27	0.003	1.23	1.08	1.41	<0.05
ECOG	3.03	1.23	7.44	0.016	2.11	0.75	5.93	0.158

*ALT, alanine aminotransferase; AST, aspartate aminotransferase; INR, international normalized ratio; WBC, White Blood Cell; PLT, Platelet; HB, Hemoglobin; MELD, Model for End-stage Liver Disease; PVT, portal vein thrombosis. ECOG, Eastern Cooperative Oncology Group*.

† *CI, Confidence interval*.

### Post-operative Complications and Shunt Dysfunction

**Figure 2:**
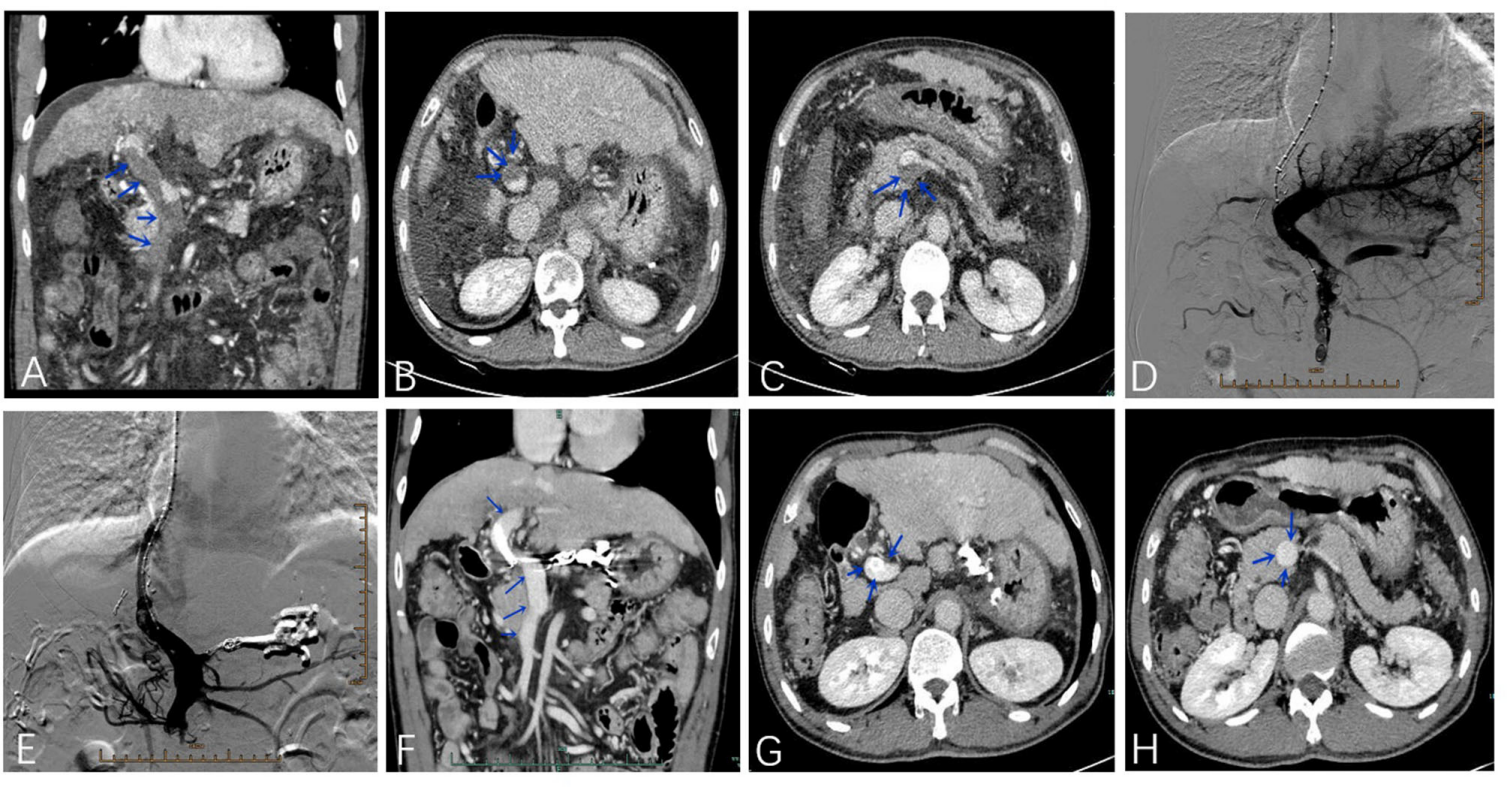
A 55-year-old male patient was treated with TIPS due to esophagogastric varices bleeding with PVT in the main portal vein and superior mesenteric vein **(A–C)**. The portal venogram before **(D)** and after **(E)** stent placement. Four months after the operation, the thrombosis disappeared in the main portal vein and superior mesenteric vein **(F–H)**.

Figure 2 presents an example of a patient with PVT in the main portal vein, and the superior mesenteric vein was treated with TIPS. All 203 patients successfully accepted TIPS between January 2015 and January 2018. Variceal bleeding (gastric plus esophageal) was the main indication for TIPS, accounting for 97.2 and 87.0% of patients with and without PVT, respectively (*P* = 0.018). The PSG was 25.3 ± 5.6 mmHg before TIPS and 10.5 ± 5.8 mmHg after TIPS. The PSG values in patients with and without PVT were similar before (*P* = 0.234) and after TIPS establishment (*P* = 0.432). The diameter of the stent required to achieve the desired reduction in the PSG was not significantly different between the PVT and no-PVT groups (*P* = 0.622). The main complications of TIPS, including recurrent variceal bleeding, recurrent ascites, and overt HE, were similar between the two groups (16.7 vs. 16.7%, *P* = 0.998; 2.7 vs. 3.8%, *P* = 0.678; and 19.4 vs. 22.9%, *P* = 0.632, respectively) (Table 2).

**Figure 3:**
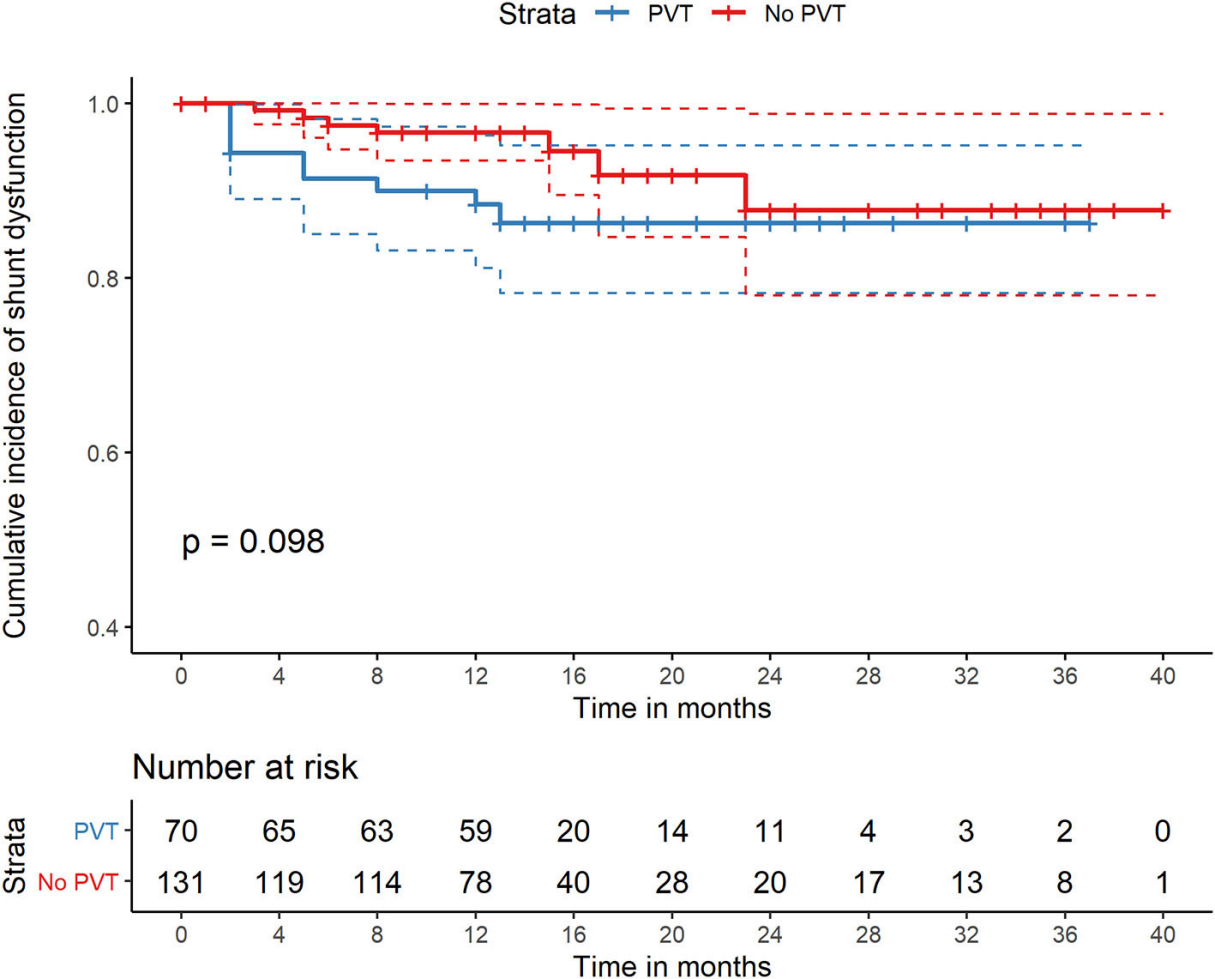
Comparison of cumulative incidence of shunt dysfunction of patients with PVT and without PVT who were all treated with TIPS. PVT, portal vein thrombosis; TIPS, transjugular intrahepatic portosystemic shunt.

During the follow-up period, 15 (7.4%) patients reported one or more episodes of shunt dysfunction. No significant difference in the cumulative incidence of shunt dysfunction was noted between the two groups during the follow-up period (9.7 vs. 6.7%, *P* = 0.214) (Table 2, Figure 3). Univariate and multivariate analyses showed that 8-mm stent diameter (HR, 1.24; 95% CI, 1.09–1.41) were associated with increased shunt dysfunction risk during follow-up (Table 4).

**Table 4:** Factors associated with risk of shunt dysfunction after Transjugular intrahepatic portosystemic shunt.

**Variable**	**Univariate analysis**				**Multivariate analysis**			
	**Hazard ratio^†^(95% CI)**			***P*-value**	**Hazard ratio^†^(95% CI)**			***P*-value**
Gender (male vs. female)	0.82	0.26	2.55	0.733	0.81	0.29	2.36	0.478
Age (per year increase)	1.02	0.98	1.07	0.321	1.08	0.88	1.33	0.234
**Antiplatelet treatments (yes vs. no)**	1.19	0.44	3.20	0.735				
**History of Ascites (yes vs. no)**	0.71	0.26	1.91	0.495				
**History of diabetes (yes vs. no)**	0.40	0.05	3.01	0.372				
**Previous splenectomy (yes vs. no)**	1.19	0.44	3.20	0.735				
**Refractory Ascites (yes vs. no)**	1.22	0.28	5.38	0.791				
Varices bleeding (Gastric + Esophageal) (yes vs. no)	0.41	0.15	1.08	0.072	0.69	0.30	1.59	0.381
**PTVE**
Coil	0.66	0.14	3.16	0.607				
Glue	0.31	0.08	1.18	0.087				
Glue+Coil	0.48	0.13	1.80	0.275				
**Diameter of stent (8 vs. 10 mm)**	1.32	1.08	1.61	0.0012	1.24	1.09	1.41	0.023
Total bilirubin	0.96	0.91	1.02	0.151				
INR	0.47	0.04	5.78	0.554				
Creatinine	1.01	0.99	1.02	0.490				
PLT	1.01	1.00	1.01	0.074	1.00	0.99	1.01	0.587
WBC	0.99	0.88	1.11	0.851				
HB	1.01	0.99	1.03	0.491				
Child	0.97	0.68	1.38	0.854				
MELD	0.93	0.76	1.13	0.445				
ECOG	0.79	0.58	1.08	0.352				

*ALT, alanine aminotransferase; AST, aspartate aminotransferase; INR, international normalized ratio; WBC, White Blood Cell; PLT, Platelet; HB, Hemoglobin; MELD, Model for End-stage Liver Disease; PVT, portal vein thrombosis. ECOG, Eastern Cooperative Oncology Group*.

† *CI, Confidence interval*.

## Discussion

In the past, PVT was considered a relative contraindication to TIPS; however, many previous studies have shown that similar outcomes were reported in patients with non-oncologic PVT and those without PVT after the creation of TIPS (13, 19, 20). A recent systematic review and meta-analysis suggested that TIPSs in patients with PVT yielded satisfactory outcomes. For example, the 1-year portal vein recanalization rate was 77.7%, the TIPS patency rate was 84.2%, and the overall 1-year survival was 87.4% (21). The results from our retrospective study also suggest that TIPS represents an alternative for the treatment of refractory ascites and variceal bleeding (gastric plus esophageal) in patients with cirrhosis with PVT. These findings based on the results of our study revealed no statistically significant difference in recurrent variceal bleeding, recurrent ascites, OHE, or shunt dysfunction between the PVT and no-PVT groups.

The mortality, complication, and shunt dysfunction rates reported in our study align with those reported in previous studies (22, 23). Our results indicate that age, MELD score, and refractory ascites requiring TIPS were risk factors for mortality in multivariate analysis. PVT is not a risk factor for mortality after TIPS. Comparisons of high-risk factors for mortality have revealed differences in some epidemiological data (age, MELD score, and refractor ascites). Mortality after TIPS increases, and this finding is closely related to increasing MELD scores, which is consistent with that reported in previous literature (23, 24). PVT increased the risk of variceal rebleeding in patients with cirrhosis (25), especially in cases of portal cavernoma and acute PVT with an increase in portal hypertension, which may cause life-threatening acute refractory variceal bleeding in those with refractory ascites (26). Thus, these patients might be at a high risk and receive benefits from TIPS insertion *via* resolution of the thrombosed portal vein and simultaneous reductions in the PSG. Furthermore, our results demonstrated that refractory ascites before TIPS may be associated with poor mortality. We believe there may be one possible explanation for this finding. The refractory ascites requiring TIPS, criteria for patient selection, can improve survival, as previously reported in the literature (3). For example, most patients were <65 years old, classified with an early stage (Child B) of disease, and did not experience prior encephalopathy, which might partially explain these excellent results (27). However, most of the patients with refractory ascites requiring TIPS in our entire cohort were classified as Child C or were elderly patients. In addition, the number of patients in this study was small, and so these patients may have had worse mortality. Our results showed that 15 (7.4%) of the entire cohort experienced shunt dysfunction, and this prevalence is lower than results from previous studies (23).

Multivariate analysis showed that smaller stent diameters were associated with increased shunt dysfunction risk in the follow-up, which is consistent with previous studies (25). The choice of stent diameter (8 vs. 10 mm) remains controversial in the literature, and there is no consensus. A previous clinical trial suggested that the use of 8-mm diameter stents for TIPS construction leads to unsatisfactory control of portal hypertension with recurrence or persistence of complications in the majority of patients (28). However, a recent clinical trial suggested that TIPS with 8-mm diameter covered stents showed similar shunt function to TIPS with 10-mm diameter stents (29). In addition, recent studies have revealed that a smaller 8-mm diameter (V. S 10-mm diameter) TIPS stent graft appears to improve patient outcomes, such as survival (30, 31). The inconsistent findings may be due to the heterogeneity of patients and the small sample size in different studies. Therefore, further clinical trials on this topic based on restricted inclusion criteria and larger sample sizes are warranted.

Our study reveals that the usage of glue in PTVE may improve overall survival and prevent shunt dysfunction, which may be preferable over coil embolization alone. This finding was consistent with the results of a recent study (32). One explanation for this finding may be that, due to the physical properties of the glue embolization material, it may propagate more readily and thoroughly into the network of PTVE, thus, leading to a cast-like formation accumulating in the periphery of PTVE. However, in practice it is more challenging to use fluid embolism materials, particularly glue, and these are more prone to off-target embolism. Coils are more precise and easier to apply, especially if removable coils are used. In addition, the cost for glue and coils (pushable and detachable) may vary in different countries. Therefore, the decision for either embolization method needs to take these regional conditions into account.

Overt hepatic encephalopathy post-TIPS occurred in 21.6% of patients in our study, and this finding is consistent with the literature (20, 33). Our results also demonstrated that PVT is not related to the incidence of OHE after TIPS. Furthermore, no patient who required a small shunt diameter developed refractory hepatic encephalopathy, and this finding may be because we excluded patients with spontaneous or recent HE. However, a randomized controlled trial (RCT) (34) suggested that either lactitol or rifaximin was not able to prevent post-TIPS encephalopathy. We still routinely prescribed lactulose and/or lactic acid powder to all patients post-TIPS to reduce the time of feces in the gut. To date, post-TIPS OHE remains a problem associated with the use of ePTFE-covered stents (19). In the future, multidisciplinary cooperative analyses will be required to identify a better method to prevent and treat post-TIPS OHE. Our study also revealed that the rates of recurrent variceal bleeding and recurrent ascites after TIPS creation were similar to those reported in previous literature (35, 36). No difference was noted between the PVT group and the no-PVT group. Patients with TIPS and PVT in principle still received antiplatelet treatment in our study useless they were evaluated as having a high risk of rebleeding.

In total, 15 (7.4%) patients (seven (9.7%) in the PVT group and eight (6.1%) in the no-PVT group) cohort exhibited shunt dysfunction, and the cumulative incidence of shunt dysfunction among the entire cohort in the overall follow-up period was not significantly different between the PVT and no-PVT groups. This percentage is less than that reported for covered stents in patients with cirrhosis with or without PVT in the literature (37). In total, fifteen patients with shunt dysfunction were successfully treated, including eight patients who underwent balloon angioplasty treatment with stent placement, five patients who underwent balloon angioplasty treatment without stent placement, and two patients who were treated with parallel TIPS. As expected, an increased rate of portal vein recanalization was observed in our entire cohort, which may be largely due to the increased flow velocity established by TIPS as it promotes mechanical lysis of residual non-occlusive thrombi (the so-called “washout effect”) (19, 20, 38, 39).

There are several limitations of our study. First, our study was a retrospective analysis, which was conducted in a single center with a limited sample size. The majority of included patients had hepatitis B-related cirrhosis. Therefore, the generalization of our findings to other settings, especially Western countries, is difficult. International, multicenter, and large-sample studies may be needed in the future to better understand the effectiveness of TIPs. Second, the epidemiological features noted in the large proportion of patients in our study included HBV-related cirrhosis, which may limit the generalizability of the findings to patients with cirrhosis for other reasons. Third, an ePTFE-covered stent combined with a bare stent was implanted during the TIPS procedure rather than a Viatorr-covered stent that was not available in China during the study period. Fourth, patients with acute bleeding during TIPS creation require urgent treatment. However, acute bleeding is an indication for emergency TIPS, which may lead to differences in procedural urgency.

In conclusion, our study shows that TIPS is feasible in the management of cirrhosis with PVT. No significant differences in survival and shunt dysfunction were noted between the PVT and no-PVT groups. Age, MELD score, and refractory ascites requiring TIPS were risk factors for mortality. A history of diabetes, PTVE, 8-mm diameter stent, and PLT increase the risk of shunt dysfunction. The occurrence of recurrent variceal bleeding, recurrent ascites, and OHE was similar between the two groups. Therefore, TIPS could be considered an alternative treatment option in cirrhosis patients with PVT.

## Data Availability Statement

The raw data supporting the conclusions of this article will be made available by the authors, without undue reservation.

## Ethics Statement

This retrospective study has been reviewed and approved by the Ethics Committee in The First Affiliated Hospital, Zhejiang University School of Medicine. The patients/participants provided their written informed consent to participate in this study. Written informed consent was obtained from the individual(s) for the publication of any potentially identifiable images or data included in this article.

## Author Contributions

H-LW and J-HS designed the study. W-JL, Y-LZ, C-HN, T-YZho, and G-HZ collected the data. H-LW, T-YZhu, B-QW, S-QC, Z-NY, and LJ analyzed the data. H-LW, W-JL, and J-HS wrote the paper with input from all authors.

## Funding

The present work was funded by the Zhejiang Provincial Natural Science Foundation of China (Grant No. LZ18H180001), the National Natural Science Foundation of China (Grant No. 81971713), the National S&T Major Project of China (No. 2018ZX10301201), Grant from the Health Commission of Zhejiang Province (JBZX-202004), the Research Unit of Collaborative Diagnosis and Treatment For Hepatobiliary and Pancreatic Cancer, the Chinese Academy of Medical Sciences (2019RU019), the Key Research Development Program of Zhejiang province (Grant No. 2018C03018), the Key Science and Technology Program of Zhejiang province (No. WKJ-ZJ-1923), and the National Key R&D Program of China (No. 2017YFC0114102).

## Conflict of Interest

The authors declare that the research was conducted in the absence of any commercial or financial relationships that could be construed as a potential conflict of interest.

## Publisher's Note

All claims expressed in this article are solely those of the authors and do not necessarily represent those of their affiliated organizations, or those of the publisher, the editors and the reviewers. Any product that may be evaluated in this article, or claim that may be made by its manufacturer, is not guaranteed or endorsed by the publisher.
