# Supplementary material for: Comparison of Transjugular Intrahepatic Portosystemic Shunt in the Treatment of Cirrhosis With or Without Portal Vein Thrombosis: A Retrospective Study
Source: Front Med (Lausanne). 2021 Oct 4;8:737984. doi: 10.3389/fmed.2021.737984 (PMC8523019; doi:10.3389/fmed.2021.737984)
Supplement: Supplementary file 1 [file Data_Sheet_1.docx]

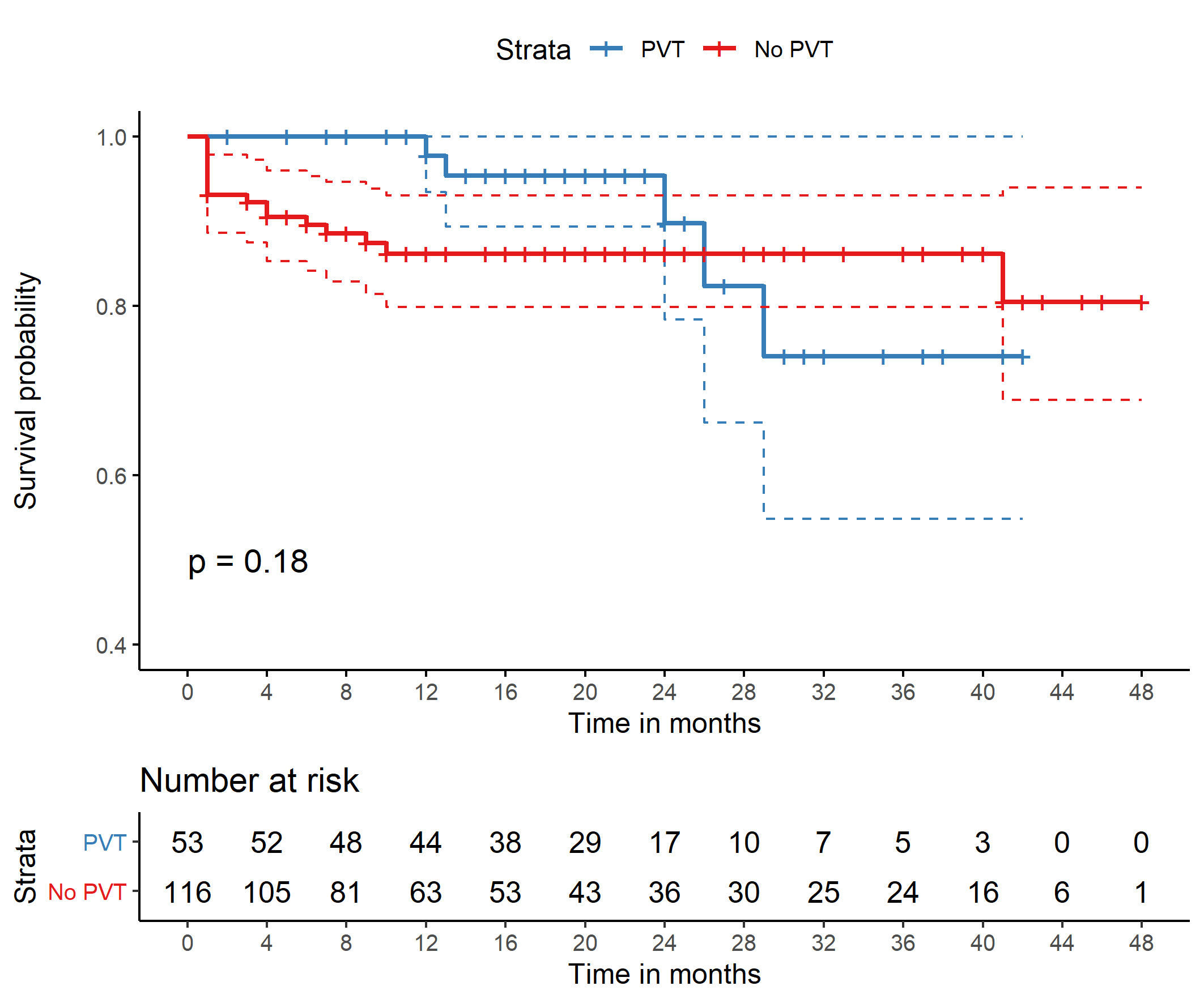


**sFigure 1.** Comparison of survival of patients with PVT and without PVT who were all treated with TIPS among patients without previous splenectomy. PVT, Portal vein thrombosis. TIPS, transjugular intrahepatic portosystemic shunt.


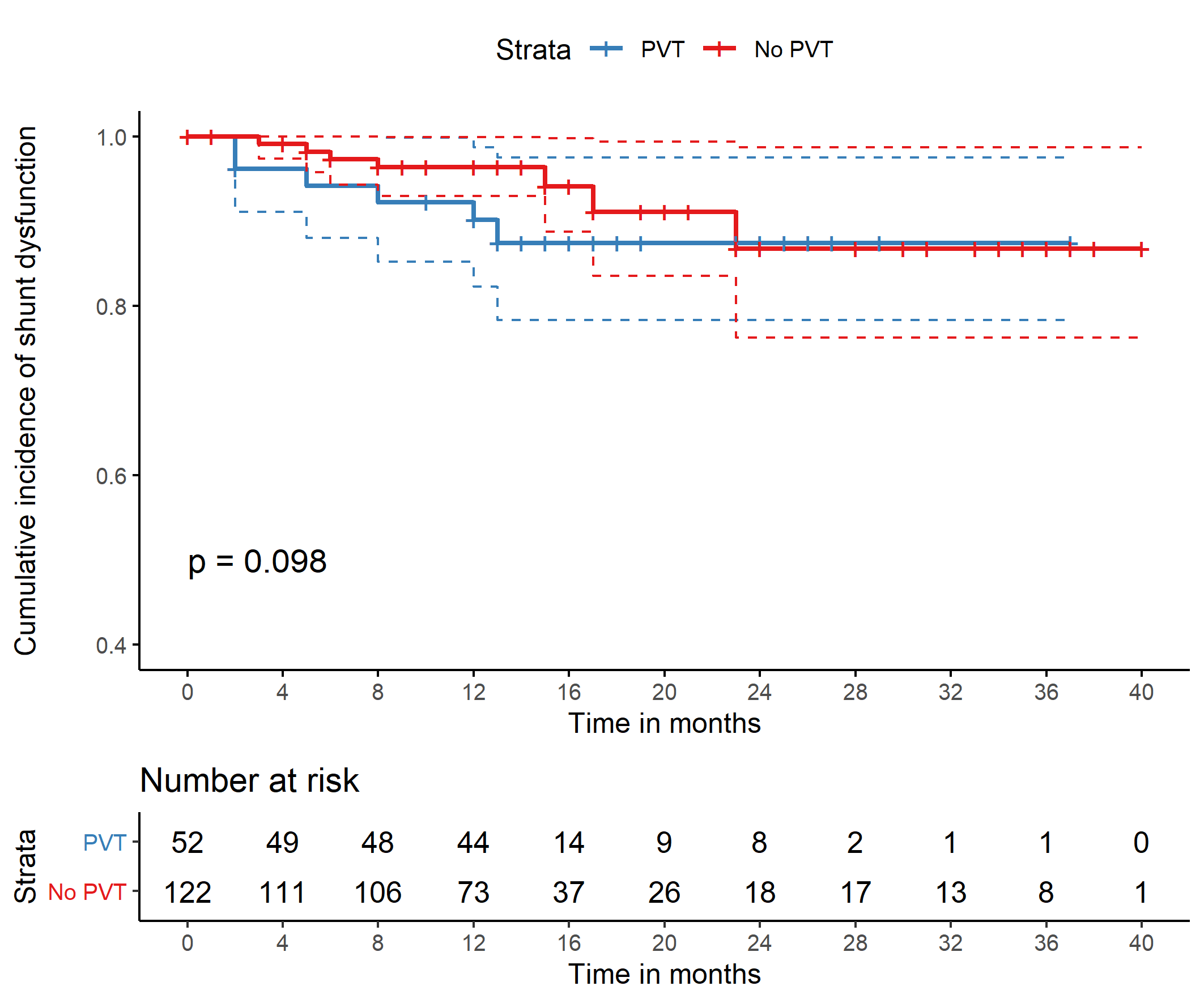


**sFigure 2.** Comparison of cumulative incidence of shunt dysfunction of patients with PVT and without PVT who were all treated with TIPS among patients without previous splenectomy. PVT, Portal vein thrombosis. TIPS, transjugular intrahepatic portosystemic shunt.

| sTable 1: Baseline Demographic and Clinical Characteristics of Patients without previous splenectomy (n=176) | | | | | |
| --- | --- | --- | --- | --- | --- |
| **Demographics** | | Overall(N=176) | PVT(N=54) | No- PVT(N=122) | P-value |
| Male | | 121(68.8%) | 33(61.1%) | 88(72.1%) | 0.201 |
| Age (mean ±SD) (years) | | 55.6±11.1 | 55.4±9.9 | 55.7±11.6 | 0.8707 |
| **Etiology of cirrhosis** | |  |  |  |  |
|  | Hepatitis B virus | 97(45.5%) | 31(57.3%) | 49(40.2%) | 0.289 |
|  | Hepatitis C virus | 4(2.3%) | 1(1.9%) | 3(2.4%) | 0.341 |
|  | Alcohol | 34(12.5%) | 8(14.7%) | 14(11.5%) | 0.655 |
|  | NASH/cryptogenic | 26(10.8%) | 7(12.9%) | 12(9.8%) | 0.211 |
|  | PBC/PSC | 7(3.9%) | 1(1.9%) | 6(4.9%) | 0.437 |
|  | Autoimmune | 5(1.7%) | 1(1.9%) | 2(1.6%) | 0.334 |
|  | Schistosome | 10(3.4%) | 1(1.9%) | 5(4.2%) | 0.273 |
| **History of diabetes** | | 24(13.6%) | 17(31.5%) | 7(5.7%) | ＜0.001 |
| **History of overt HE** | | 8(4.5%) | 2(3.7%) | 6(4.9%) | 0.115 |
| **Laboratory parameters (mean ±SD)** | |  |  |  |  |
|  | AST (IU/l) | 83.1±212 | 85.2±128 | 73.7±176 | 0.323 |
|  | ALT (IU/l) | 65.2±172 | 66.9±124 | 64.3±225 | 0.418 |
|  | Albumin (g/dl) | 34.3±5.5 | 34.0±4.3 | 34.5±6.0 | 0.579 |
|  | INR | 1.3±0.2 | 1.3±0.2 | 1.3±0.2 | 0.852 |
|  | WBC | 5.1±5.4 | 6.6±8.6 | 4.4±2.8 | 0.068 |
|  | PLT | 79.7±63.6 | 117.6±86.7 | 62.9±40.4 | ＜0.001 |
|  | HB | 82.4±24.6 | 78.4±22.6 | 84.2±25.3 | 0.128 |
|  | Creatinine (mg/dl) | 71.3±34.7 | 65.4±16.9 | 74.0±40.0 | 0.049 |
|  | Total bilirubin (mg/dl) | 21.9±14.4 | 21.8±15.9 | 22.0±13.8 | 0.944 |
| **Child-Pugh score** | | 6.7±1.3 | 6.8±1.3 | 6.7±1.4 | 0.775 |
| **Child-Pugh class** | |  |  |  | 0.543 |
|  | A | 76(43.2%) | 24(44.4%) | 52(42.6%) |  |
|  | B | 86(48.9%) | 34(62.9%) | 52(42.6%) |  |
|  | C | 12(6.8%) | 3(5.5%) | 9(7.4%) |  |
| **MELD** | | 10.8±3.0 | 10.6±3.0 | 10.9±3.1 | 0.525 |
| **ECOG** | | 1.08±0.35 | 1.09±0.35 | 1.07±0.34 | 0.742 |
| **Stage of PVT (chronic)** | | / | 43(79.6%) | / |  |
| **Degree of PVT** | | / |  | / |  |
| Mural | | / | 8(14.8%) | / |  |
| Partial | | / | 48(88.0%) | / |  |
| Complete | | / | 3(5.5%) | / |  |
| **Extent of PVT** | | / |  | / |  |
| MPV alone | | / | 10(18.5%) | / |  |
| MPV + SMV | | / | 15(27.7%) | / |  |
| MPV + SV/splenectomy | | / | 27(50.0%) | / |  |
| MPV + SMV + SV/splenectomy | | / | 5(9.2%) | / |  |
| NASH, nonalcoholic steatohepatitis; PBC, primary biliary cirrhosis; PSC, primary sclerosing cholangitis; HE, hepatic encephalopathy; ALT, alanine aminotransferase; AST, aspartate aminotransferase; INR, international normalized ratio; WBC, White Blood Cell; PLT, Platelet; HB, Hemoglobin; MELD, Model for End-stage Liver Disease; PVT, portal vein thrombosis. ECOG, Eastern Cooperative Oncology Group; MPV, main portal vein; SMV, superior mesenteric vein; SV, splenic vein. | | | | | |

| **sTable 2.** Outcomes of Transjugular intrahepatic portosystemic shunt in patients without previous splenectomy(n=176) | | | | | |
| --- | --- | --- | --- | --- | --- |
| **Characteristics** | | Overall(N=176) | PVT(N=54) | No- PVT(N=122) | P-value |
| **Duration of follow-up (month)** | | 18.7±11.8 | 19.5±9.2 | 18.6±12.2 | 0.124 |
| **Indications for TIPS** | |  |  |  |  |
|  | Refractory Ascites | 16(9.1%) | 8(14.8%) | 8(6.6%) | 0.141 |
|  | variceal bleeding (Gastric+Esophageal) | 121(68.8%) | 33(61.1%) | 88(72.1%) | 0.201 |
| **90-Day Mortality** | | 25(14.2%) | 6(11.1%) | 19(15.6%) | 0.584 |
| **Mortality** | | 17(9.7%) | 4(7.5%) | 13(10.6%) | 0.118 |
|  | Liver failure | 6(3.4%) | 2(3.7%) | 4(3.3%) |  |
|  | Multiorgan failure | 4(2.3%) | 1(1.8%) | 3(2.5%) |  |
|  | Gastrointestinal bleeding | 2(1.1%) | 1(1.8%) | 1(0.8%) |  |
|  | Hepatorenal syndrome | 0(0%) | 0(0.0%) | 0(0%) |  |
|  | Sepsis | 1(0.6%) | 0(0.0%) | 1(0.8%) |  |
|  | Cerebral hemorrhage | 0(0%) | 0(0.0%) | 0(0%) |  |
|  | Unknown | 2(1.1%) | 1(1.8%) | 1(0.8%) |  |
| **Diameter of stent** | |  |  |  | 1.000 |
|  | 8mm | 164(93.2%) | 55(92.6%) | 109(93.4%) |  |
|  | 10mm | 12(6.8%) | 4(7.4%) | 8(6.6%) |  |
| **Overt hepatic encephalopathy** | | 33(19.8%) | 11(20.4%) | 22(18%) | 0.432 |
| **Recurrent variceal bleeding** | | 28(16.7%) | 10(18.5%) | 18(14.7%) | 0.321 |
| **Recurrent ascites** | | 3(1.8%) | 1(1.7%) | 3(2.5%) | 0.338 |
| **Antiplatelet treatments** | | 85(51.2%) | 42(77.7%) | 43(35.2%) | <0.01 |
|  | Aspirin | 60(35.9%) | 28(51.8%) | 32(36.8%) | 0.011 |
|  | Aspirin+Dipyridamole | 27(16.2%) | 10(18.5%) | 17(13.9%) | 0.632 |
| **PTVE** | | 120(68.2%) | 33(27.5%) | 87(72.5%) | 0.661 |
|  | Coil | 61(50.8%) | 19(57.6%) | 42(48.3%) |  |
|  | Glue | 17(14.2%) | 4(12.1%) | 13(14.9%) |  |
|  | Glue+Coil | 42(35.0%) | 10(30.3%) | 32(36.8%) |  |
| **Portosystemic gradient before TIPS (mmHg)** | | 24.2±5.2 | 26.4±6.1 | 23.1±4.3 | 0.193 |
| **Portosystemic gradient after TIPS** | | 11.2±5.1 | 11.2±3.8 | 9.2±3.5 | 0.556 |
| **Shunt dysfunction** | | 13(7.4%) | 6(11.1%) | 7(5.7%) | 0.345 |
| Indications for TIPS, 90-Day Mortality, Mortality, Diameter of stent, Overt hepatic encephalopathy, Recurrent variceal bleeding, Recurrent ascites, Antiplatelet treatments, PTVE, PSG before TIPS(mmHg), PSG after TIPS(mmHg),Liver transplantation, Shunt dysfunction compared between patients with and without PVT.  OHE, Overt hepatic encephalopathy PSG, Portosystemic gradient, PTVE percutaneous transhepatic variceal embolization. | | | | | |

| **sTable 3** Factors Associated with Risk of mortality after Transjugular intrahepatic portosystemic shunt in patients without previous splenectomy(n=176) | | | | | | | | | |
| --- | --- | --- | --- | --- | --- | --- | --- | --- | --- |
| Variable | Univariate Analysis | | | |  | Multivariate Analysis | | | |
|  | Hazard Ratio† (95% CI) | | | P Value |  | Hazard Ratio†(95% CI) | | | P Value |
| Gender (male vs. female) | 1.68 | 0.71 | 4.00 | 0.241 |  | 1.06 | 0.40 | 2.82 | 0.905 |
| Age (per year increase) | 1.06 | 1.02 | 1.11 | 0.003 |  | 1.05 | 1.00 | 1.10 | ＜0.05 |
| **Antiplatelet treatments (yes vs. no)** | 0.40 | 0.16 | 1.00 | 0.049 |  | 0.48 | 0.19 | 1.25 | 0.132 |
| **History of Ascites** (yes vs. no) | 0.94 | 0.39 | 2.29 | 0.892 |  |  |  |  |  |
| **History of diabetes (yes vs. no)** | 0.56 | 0.13 | 2.43 | 0.443 |  |  |  |  |  |
| **Refractory Ascites (yes vs. no)** | 0.5 | 0.31 | 0.81 | ＜0.05 |  | 0.46 | 0.28 | 0.74 | ＜0.05 |
| **Varices bleeding (Gastric+Esophageal) (yes vs. no)** | 0.57 | 0.24 | 1.36 | 0.207 |  |  |  |  |  |
| **PTVE ( vs. no PTVE)** |  |  |  |  |  |  |  |  |  |
| Coil | 0.74 | 0.27 | 2.00 | 0.554 |  |  |  |  |  |
| Glue | 0.26 | 0.03 | 2.12 | 0.210 |  |  |  |  |  |
| Glue+Coil | 0.57 | 0.17 | 1.85 | 0.347 |  |  |  |  |  |
| **Diameter of stent (8mm vs. 10mm)** | 0.63 | 0.23 | 1.72 | 0.366 |  |  |  |  |  |
| Total bilirubin | 1.00 | 0.97 | 1.03 | 0.863 |  |  |  |  |  |
| INR | 1.56 | 0.23 | 10.75 | 0.650 |  |  |  |  |  |
| Creatinine | 1.01 | 1.01 | 1.02 | ＜0.001 |  | 1.01 | 1.00 | 1.02 | 0.119 |
| PLT | 1.00 | 0.99 | 1.01 | 0.693 |  |  |  |  |  |
| WBC | 0.98 | 0.89 | 1.09 | 0.704 |  |  |  |  |  |
| HB | 1.00 | 0.98 | 1.02 | 0.955 |  |  |  |  |  |
| Child | 1.33 | 0.99 | 1.77 | 0.056 |  | 1.20 | 0.79 | 1.84 | 0.393 |
| MELD | 1.20 | 1.08 | 1.34 | ＜0.001 |  | 1.32 | 1.18 | 1.48 | <0.01 |
| ECOG | 2.43 | 0.87 | 6.79 | 0.091 |  | 1.40 | 0.41 | 4.81 | 0.589 |
| ALT, alanine aminotransferase; AST, aspartate aminotransferase; INR, international normalized ratio; WBC, White Blood Cell; PLT, Platelet; HB Hemoglobin; MELD, Model for End-stage Liver Disease; PVT, portal vein thrombosis. ECOG, Eastern Cooperative Oncology Group. | | | | | | | | | |

| **sTable 4** Factors Associated with Risk of shunt dysfunction after Transjugular intrahepatic portosystemic shunt in patients without previous splenectomy(n=176) | | | | | | | | | |
| --- | --- | --- | --- | --- | --- | --- | --- | --- | --- |
| Variable | Univariate Analysis | | | |  | Multivariate Analysis | | | |
|  | Hazard Ratio† (95% CI) | | | P Value |  | Hazard Ratio†(95% CI) | | | P Value |
| Gender (male vs. female) | 1.06 | 0.33 | 3.45 | 0.922 |  | 0.80 | 0.23 | 2.81 | 0.731 |
| Age (per year increase) | 1.03 | 0.98 | 1.09 | 0.192 |  | 1.04 | 0.98 | 1.09 | 0.196 |
| **Antiplatelet treatments (yes vs. no)** | 1.06 | 0.35 | 3.16 | 0.919 |  |  |  |  |  |
| **History of Ascites** (yes vs. no) | 0.59 | 0.20 | 1.74 | 0.336 |  |  |  |  |  |
| **History of diabetes (yes vs. no)** | 0.49 | 0.06 | 3.80 | 0.497 |  |  |  |  |  |
| **Refractory Ascites (yes vs. no)** | 0.85 | 0.11 | 6.51 | 0.873 |  |  |  |  |  |
| **Varices bleeding (Gastric+Esophageal) (yes vs. no)** | 0.34 | 0.12 | 1.02 | 0.055 |  | 0.35 | 0.12 | 1.06 | 0.063 |
| **PTVE ( vs. no PTVE)** |  |  |  |  |  |  |  |  |  |
| Coil | 0.24 | 0.05 | 1.14 | 0.073 |  |  |  |  |  |
| Glue | 0.35 | 0.04 | 2.88 | 0.327 |  |  |  |  |  |
| Glue+Coil | 0.57 | 0.15 | 2.22 | 0.418 |  |  |  |  |  |
| **Diameter of stent (8mm vs. 10mm)** | 1.44 | 1.11 | 1.87 | <0.01 |  | 1.61 | 1.12 | 2.31 | 0.012 |
| Total bilirubin | 0.98 | 0.93 | 1.04 | 0.515 |  |  |  |  |  |
| INR | 0.63 | 0.04 | 11.07 | 0.749 |  |  |  |  |  |
| Creatinine | 1.01 | 0.99 | 1.02 | 0.401 |  |  |  |  |  |
| PLT | 1.00 | 1.00 | 1.01 | 0.327 |  |  |  |  |  |
| WBC | 1.01 | 0.92 | 1.10 | 0.874 |  |  |  |  |  |
| HB | 1.01 | 0.99 | 1.03 | 0.542 |  |  |  |  |  |
| Child | 1.06 | 0.70 | 1.60 | 0.786 |  |  |  |  |  |
| MELD | 0.98 | 0.79 | 1.21 | 0.839 |  |  |  |  |  |
| ECOG | 1.26 | 0.78 | 2.06 | 0.498 |  |  |  |  |  |
| ALT, alanine aminotransferase; AST, aspartate aminotransferase; INR, international normalized ratio; WBC, White Blood Cell; PLT, Platelet; HB Hemoglobin; MELD, Model for End-stage Liver Disease; PVT, portal vein thrombosis. ECOG, Eastern Cooperative Oncology Group. | | | | | | | | | |
